# The race/color question in the care process in a psychosocial care center for children and adolescents

**DOI:** 10.1590/1980-220X-REEUSP-2021-0363

**Published:** 2022-03-14

**Authors:** Bruna de Paula Candido, Sônia Barros, Luciane Régio, Caroline Ballan, Márcia Aparecida Ferreira de Oliveira

**Affiliations:** 1 Universidade de São Paulo, Escola de Enfermagem, Programa de pós-graduação em Enfermagem, São Paulo, SP, Brazil.; 2 Universidade de São Paulo, Escola de Enfermagem, Departamento de Enfermagem Materno Infantil e Psiquiátrica, São Paulo, SP, Brazil.; 3 Universidade de São Paulo, Escola de Enfermagem da USP, Programa de Doutorado Interunidades, São Paulo, SP, Brazil.; 4 Universidade de São Paulo, Instituto de Estudos Avançados, São Paulo, SP, Brazil.

**Keywords:** Community Mental Health Centers, Child, Adolescent, Racism, Intersectoral Collaboration, Centros Comunitarios de Salud Mental, Niño, Adolescente, Racismo, Colaboración Intersectorial, Centros Comunitários de Saúde Mental, Criança, Adolescente, Racismo, Colaboração Intersetorial

## Abstract

**Objective:**

To recognize the actions related to race/color developed in the care process of the black child and adolescent population of a Psychosocial Care Center for children and adolescents in Brazil.

**Method:**

This is a study with a qualitative approach focusing on the three-dimensional racism framework. Data were collected through active medical records and interviews with reference professionals. The results were categorized and thematized through content analysis and the following themes were found: typology of child violence, identification of racism, the school, access to black culture and representativeness. This study obtained ethical approval.

**Results:**

The race/color question in the face of violation of fundamental rights of black children/adolescents contributes to the understanding of racism as a social determinant of mental health. Actions to empower the black population include the insertion of the race-color question as an analytical and procedural category in the Singular Therapeutic Projects, as an integral practice of multiprofessional teams work process.

**Conclusion:**

It is necessary to invest persistently in the identification and qualification of actions and systematic discussions to face the psychosocial effects of racism.

## RESUMO

**Objetivo:**

Reconhecer as ações relacionadas ao quesito raça/cor desenvolvidas no processo de cuidado da população infantojuvenil negra de um Centro de Atenção Psicossocial infantojuvenil no Brasil.

**Método:**

Pesquisa de abordagem qualitativa com enfoque no referencial do racismo tridimensional. Os dados foram coletados por meio dos prontuários ativos e entrevistas com profissionais de referência. Os resultados foram categorizados e tematizados por meio de análise de conteúdo e encontraram-se os seguintes temas: tipologia da violência infantil, identificação do racismo, a escola, acesso à cultura negra e representatividade. Este estudo obteve aprovação ética.

**Resultados:**

O quesito raça/cor frente à violação de direitos fundamentais da criança/adolescente negro contribui para a compreensão do racismo enquanto determinante social de saúde mental. As ações de empoderamento da população negra perpassam pela inserção do quesito raça-cor como categoria analítica e processual nos Projetos Terapêuticos Singulares, como prática integrante do processo de trabalho das equipes multiprofissionais.

**Conclusão:**

É preciso investir persistentemente na identificação e na qualificação de ações e discussões sistemáticas para enfrentar os efeitos psicossociais do racismo.

## RESUMEN

**Objetivo:**

Reconocer las acciones relacionadas a la cuestión raza/color desarrolladas en el proceso de cuidado de la población infantojuvenil negra de un Centro de Atención Psicosocial infantojuvenil en Brasil (CAPSij).

**Método:**

Investigación de abordaje cualitativo con enfoque en el referencial del racismo tridimensional. Los datos fueron recolectados a través de los historiales activos y entrevistas con profesionales de referencia. Los resultados fueron categorizados y tematizados por medio de análisis de contenido y se hallaron los siguientes temas: tipología de la violencia infantil, identificación del racismo, la escuela, acceso a la cultura negra y representatividad. Este estudio obtuvo aprobación ética.

**Resultados:**

La cuestión raza/color frente a la violación de derechos fundamentales del niño/adolescente negro contribuye para la comprensión del racismo como determinante social de salud mental. Las acciones de empoderamiento de la población negra abarcan la inserción de la cuestión raza/color como categoría analítica y procesual en los Proyectos Terapéuticos Singulares, como práctica integrante del proceso de trabajo de los equipos multiprofesionales.

**Conclusión:**

Es necesario invertir persistentemente en la identificación y en la cualificación de acciones y debates sistemáticos para enfrentar los efectos psicosociales del racismo.

## INTRODUCTION

After the promulgation of the 1988 Federal Constitution, children and adolescents became citizens with rights, detailed in Law No. 8.069/90, which originated the Child and Adolescent Statute (*ECA*). Children and adolescents are holders of fundamental rights inherent to human beings, including the right to dignity. Article 3 of *ECA* assigns to everyone the duty to respect this population with the highest priority, putting them safe from any form of discrimination or oppression, including racial discrimination^(1,2,3)^.

The current inequities regarding access to rights experienced by the black population have their roots in the colonial structure of exploitation, violence, exclusion, epistemicide, and extermination of values and traditions of indigenous peoples, which leads to complex marginalization, social inequalities, and vulnerabilities^(4)^.

The difficulty of creating guidelines to combat racism in Brazil is based on the myth of racial democracy, in the denial of its existence, with extreme resistance to the naming of this social phenomenon, which establishes another, called whiteness. Its definition is the position of systematized privileges, of “material and symbolic resources, initially generated by colonialism and imperialism, and that are maintained and preserved in contemporary times”, linking racism in Brazil “to status and phenotype”^(5, p.84)^.

Historically, black people have been and continue to be excluded due to racism, with a significant loss of contractual value, a condition that can be superimposed when the person has mental health problems, with a movement from enslavement to asylum being observed^(6)^.

The advances achieved by the Brazilian Psychiatric Reform (*RPB*) were expressed and based on Law No. 10.216/2001, through the guarantee of protection and rights, formalization of the psychosocial care model in the national territory. However, the discussion about the impact of racism on people’s mental health is recent^(7)^.

According to a study, there was a late construction of the child and adolescent perspective in the Mental Health Policy. It was at the II National Conference on Mental Health that the rights of children and adolescents were recognized and the agenda of mental health care for children and adolescents was included in Ordinance No. 336/2002, with *CAPSi* as a specific modality^(8)^.

To minimize social inequalities and to understand racism as a social determinant in the health/disease process, the National Comprehensive Health Policy for the Black Population (*PNSIPN*) was created^(9)^. In the same way, psychosocial care recognized intersectoriality as a strategy to guarantee rights and focused on the organization of the Psychosocial Care Network (*RAPS*)^(10)^. Also to face inequalities, in 2015, Ordinance No. 1.130 instituted the National Policy for Comprehensive Care for Children’s Health (*PNAISC*) within the scope of the Brazilian Public Health System (*SUS*)^(11)^.

However, as of 2016, the country is “taken into the past”. Thus, *SUS* and *RPB* undergo several setbacks, starting with the approval of the constitutional amendment EC 95/2016, which establishes the freezing of investments in the areas of health and education for 20 years. A period begins that worsens *SUS* underfunding, disregards the establishment of the right to health, and the growing trajectory of expansion of health care networks, and takes the path of State disinvestment in social areas^(12)^.

In the radicalization of regressive mental health and human rights policies, eugenic ideals are updated in necropolitics established in Brazil, a policy of symbolic death and real extermination of black people, and so the asylums go beyond their walls^(13)^.

In the psychosocial field, the object of care is the person, according to Basaglia, and it is the disease that needs to be in parentheses. To deinstitutionalize the paradigm is to guide relationships and actions from the perspective of citizenship with rights, freedom, fight against racism, to strengthen community life, social support networks, to invest in mental health workers so that they are agents of social transformation^(14)^.

In freedom, care serves people, so that they are protagonists of their lives and stories, expanding autonomy. Beyond institutional walls, it is ethical responsibility to interfere with the stigma under the “definition of social norm and of who the subjects considered deviant are” ^(8, p.202)^.

Assuming that black children and adolescents suffer double discrimination, due to the stigma of madness and skin color, this study focuses on how the service faces the psychosocial effects of racism, recognizing the children’s CAPS as citizenship devices, capable of acting to confront these inequities^(15)^. Thus, the research question is: do the actions developed in the CAPS for children and adolescents, based on the race/color question, allow or not the social inclusion of black children and adolescents with mental health problems? The aim of the study was to recognize actions related to race/color questions developed by the Psychosocial Care Center for children and adolescents (*CAPSij*), in the care of black children and adolescents.

## METHOD

### Design of Study

This is a qualitative approach study^(16)^. As a theoretical framework, the three dimensions of racism proposed by Werneck were used^(17)^: (a) personal racism causes feelings of superiority and proactivity, inferiority and passivity; (b) in interpersonal racism, actions and omissions take place. There is lack of respect, distrust, devaluation, persecution, and dehumanization towards black individuals and their communities, reiterating the omissions for all these actions; there is neglect when dealing with racism and its consequences; (c) finally, institutional racism is the structural mechanism to maintain racism in society; it uses reduced material (access to quality policies) and less access to power (less access to information, less access to social control, and lack of resources); establishes barriers, legitimizing inequities and, in the author’s words, is the “most neglected dimension” and that which maintains the “vulnerability of individuals and social groups” ^(17)^.

### Local

The study setting was a *CAPS* for children and adolescents II, located in the North Zone of the city of São Paulo. Its coverage area goes from the neighborhoods Vila Brasilândia to Freguesia do Ó and has an approximate population of 420,000 inhabitants. The service follows children and adolescents up to 18 years of age with serious and persistent mental health problems.

### Population

One of the data sources were the active medical records in the 30 days prior to collection, of all children and adolescents self-declared as black or brown. The corpus consisted of 220 medical records, 175 self-declared brown and 45 black. As exclusion criteria, exits, duplication of data, non-embracements, embracement after the stipulated date for the start of collection, and race/color not declared in the medical record were used.

To complement information, interviews were carried out with Reference Technicians (TR), technical and/or higher education professionals, part of the service’s multidisciplinary team, in charge of the closest monitoring of each black child/adolescent. A pair of reference technicians (TR1 and TR2) was defined according to the initial embracement and the micro- region of residence. The selection for the interview sample was preferably for TR1, and in his/her absence, TR2 of black children and adolescents was sought. They were all oriented about the study and were given the Free and Informed Consent Term (FICT), according to CNS Resolution No. 466/2012. The interviews were carried out at *CAPSij*, with an average duration of 30 minutes.

### Data Collection

The following items were collected from the medical records: medical record number; identification of the child/adolescent (name, date of birth, age, sex); history of childhood violence (sexual, psychological, physical abuse and unspecified maltreatment); history of racism (humiliation, inferiorization, persecution, joking nicknames) in places frequented by the black child/adolescent (school, residence, *CAPSij*); history of non-acceptance of their stereotype Afro-descendant appearance and traits (skin tone, nose, hair and/or mouth); history of access to spaces and to black culture promoted by the mental health service.

In the interviews with TRs, a structured questionnaire was used and contained: identification of the reference technician (name, date of birth, age, sex); name of the child/adolescent; number of the child/adolescent medical record; history of childhood violence (sexual, psychological, physical abuse and unspecified maltreatment); history of racism (humiliation, inferiorization, persecution, joking nicknames) in places frequented by the black child/adolescent (school, residence, *CAPSij*); history of non-acceptance of their black stereotypical appearance and features (skin color, nose, hair and/or mouth); history of access to spaces and to black culture objects promoted by the mental health service.

During collection, there were 30 TRs at *CAPSij* in the multidisciplinary team; however, 22 technicians were responsible for the self-declared black or brown children/adolescents. Among the 22 TRs responsible for black children and adolescents at *CAPSij*, in the period defined for the collection, six agreed to participate in the research, that is, 27.27%. And, of these six TRs, two self-declared themselves as white, two as brown, and two as black. The interviews in this sample were carried out over a period of three days, totaling six TRs (27.27%).

### Data Analysis and Treatment

Data analysis was made through content analysis following the phases proposed by Bardin^(18)^. The following themes were found: typology of child violence, identification of racism, the school, access to black culture and representativeness. The statements by the TRs interviewed are presented with different letters of the alphabet A, B, C, D, E and F, to ensure anonymity. For the treatment and discussion of the findings, we based ourselves on the institutional racism framework, as well as on the scientific literature.

### Ethical Aspects

The study was approved by the competent agencies in 2018, by the Research Ethics Committees of the Nursing School of Universidade de São Paulo, and the Municipal Health Department of São Paulo (2.542.896/2018 e 2.638.411/2018), in accordance with Resolution no. 466/2012, of the National Health Council. All participants signed the FICF.

## RESULTS

Among the 220 medical records of users self-declared black and brown at *CAPSij*, the occurrence of child violence was included in 60 records (27%), 160 records did not have records about this (73%). The violence identified was psychological abuse (54%), physical violence (23%), followed by sexual abuse (14%), and general maltreatment (9%).

Regarding racism and the place of occurrence, the reports in medical records revealed that 9 children/adolescents (that is, 4%) suffered racism. And of these, 100% of the cases occurred at school. It was found that, in none of the cases of racism, identified and recorded in the medical records, there were coping actions described by the service, which may highlight the institutional dimension of racism in health services^(17)^.

There were also no reports identified in the medical records about stimuli for Afro games, visits to Afro-centered cultural spaces, and/or the use of black dolls within the *CAPSij*, black public figures/heroes/singers/“famous teens” among the objects used for actions in the PTS, and participation in black-themed event/party, critical actions and interventions to combat and confront racism in its personal and interpersonal dimensions^(17)^.

In the personal dimension, in relation to the acceptance of their black stereotypical appearance and features (skin color, nose, hair and/or mouth), there was a report in a medical record, in which the user referred not accepting her hair, and there were also reports of self-harm.

In the TRs’ responses, the majority reported having knowledge about the typology of child violence suffered by the child/adolescent, recognizing the history of the violence suffered and the necessary confrontation, reinforcing that the topic is debated by the team.

*He gets beaten up by his mother and is bullied at school. Also, the family shows some prejudice, some uncles call him “little sissy, little girl”.(TRA)*

The TRs had difficulties in identifying racism with the black children/adolescents for whom they are responsible for care. It should be noted that the omission in dealing with racism is defined by Werneck as an unfolding of the interpersonal dimension^(17)^:

*We suspected of her suffering racism because she doesn’t like her hair either. She asks to leave it tied up. (TRF)*

Still on racism, when the child is in early childhood, some TRs say that there is no report, given the child’s age:

*Too small to bring up the issue of racism. (TRB)*

*Never talked about racism. The child is small. Neither the mother nor the family reported any cases either. (TRE)*

The school appears as the main place where racism in the interpersonal dimension occurs with children/adolescents followed at *CAPSij*, according to the following statements:

*At school, he has already been called “dirty black” and “monkey”.(TRC)*

*The adolescent was in a fight at school. The other students started it, then called the boy a “monkey”. When the teacher intervened, she expelled the black student from the class. (TRD)*

*He suffered racism, indeed. At school, he is excluded by his peers. Also, at a specific time, his care was impaired, he didn’t shower. Classmates called him “disgusting” and “slave”. (TRF)*

As an intervention in the personal dimension of racism, a professional mentioned having used the image of a black public figure to develop the work of representativeness in a therapeutic group at *CAPSij*:

*Yes, I’ve already used photos and figures of famous people such as Lázaro Ramos and Taís Araújo, and other black actors within the theater group. (TRD)*

Among the interventions, the TRs use objects to carry out actions provided for in the Singular Therapeutic Project (*PTS*), through games developed with black children of preschool age:

*Here’s a black doll, just like a baby. We only use it with younger children. For the bigger ones, we have already used the drawing I mentioned (of African origin). (TRB)*

They also say that, in general, children/adolescents do not bring up issues about disliking their black stereotypical features, except for one case, exemplified below:

*He doesn’t like his skin color, he has already said that he feels bad about that condition and that dying would solve everything. (TRD)*

In terms of representation, the event of the Black Awareness Day in the calendar was pointed out as important:

*We have the Black Awareness week, on November 20. They like it and participate a lot! (TRA)*

*During the year, there are movements related to the black culture, such as visiting the occupation and, in November, about the Black Awareness. (TRD)*

When rescuing history with children and adolescents, the stimulus is through access to the black culture, in actions described below:

*I proposed the integration to the cartography group, the visit to the black occupation at FUNARTE, and theater on blackness promoted by CAPSi. (TRD)*

*We visited the CCJ (Cultural Youth Center) which addressed the black theme. (TRE)*

*They watched the cartoon of African origin “KIRIKU”, we created this space here at CAPS, during the Black Awareness Week. (TRA)*

## DISCUSSION

In 1996, the question race/color was included in the mortality and live births information systems, evidencing racial inequities in the black population’s living conditions^(19)^. However, the discussion about the race/color profile of the population in general is still incipient, because, even if collected, it does not fulfill the function of category of analysis in work and care processes.

Faced with the denial and violation of fundamental rights of black children/adolescents, the lack of fight against racism contributes to the deleterious effects on the child/adolescent’s subjectivity, especially when racism occurs in early childhood^(20)^.

Violence, prejudice, and discrimination cause sequelae in psychic life, as social determinants of health^(21)^. An issue of fundamental importance in the care of children and adolescents who are victims of violence is the bond, and active listening, to identify, notify, and fight violence, which appears after a long period of silence and denial. When violence come up, it is up to professionals to embrace and be attentive to the feelings that follow the report. The need to work with families and social support networks should also be noted, as well as to highlight, in the intersectoral network, that they provide assistance to children and adolescents, to ensure protection and care for the needs and rights of black children and adolescents^(22)^.

In the speeches of the professionals interviewed, there are reports on violence, discrimination, and racism against children and adolescents, although the recognition of racism as violence is not stated. Violence must be identified, reported, and investigated, and it has been the main cause of children arriving at *CAPSij* ^(15)^.

To explain the ways in which racism is found in the different fields of social life, the raciality device is proposed, through the theoretical framework, as a conceptual tool that allows us to understand racism as the structuring basis of the Brazilian society^(17)^. At the interpersonal dimension of racism, expressed in prejudice and discrimination, whether intentional or not, if there is no reflection among professionals on the meaning of the race/color question in psychosocial care, invisibility can be reproduced through omissions, or “negligence in dealing with racism and its impacts”^(17)^.

The institutional dimension “establishes the structural dimension, corresponding to organizational forms, policies, practices, and standards that result in unequal treatments and results”. This occurs “in the reduced access to quality policies”, “less access to information, less participation and social control”, implicit racial violence, practiced even in health services^(17)^.

The study shows that TRs verbalize possible cases of racism suffered inside and outside *CAPS* by children/adolescents, but do not state the importance of notifying and addressing racism as violence, a social determinant of health, a producer of suffering and possible mental health problems. Health workers involved in the monitoring of black children and adolescents, through active listening and attentive eyes, can provide a safe environment for expressing and confronting racism^(21)^.

When analyzing the information about the place of greatest occurrence of racial discrimination, the school environment stands out, a space that should provide citizenship and protection. Institutional and interpersonal racism at school, especially in early childhood, and when not fought, makes the child believe in his/her “inferiority”, resulting in deleterious effects on subjectivity related to the personal dimension^(17)^.

It should be noted that, when children in early childhood and school age experience racism, discrimination affects subjective self-esteem and worsens when, at school, these acts are not fought and/or discussed^(20)^.

Studies show the need to make a more in-depth analysis of the lack of racial representation in schools. Racism perpetrated in classrooms causes effects such as loneliness and estrangement^(23)^. The situation worsens when figures of representation and history of the black population are not included in the educational plan, leading to children/adolescents with no references in the development of identity^(20)^.

The results point to the need to expand the debate on the importance of ethnic-racial questions in the field of education. Laws no. 10.639/2003 and 11.645/2008 state the mandatory teaching of African, Afro-Brazilian and indigenous history and culture in basic, public and private education systems^(23)^.

The school is an environment that provides an intense exchange of cultures and experiences among children and adolescents from different ethnic-racial groups, the “school is the place where, in addition to school knowledge and content, values and beliefs related to race, gender and social class must be worked on”^(23, p.118)^. The school is the social institution responsible for the organization, transmission, and socialization of knowledge and culture, and according to the results presented, it reveals itself as a space in which racism presents itself. Therefore, it is also an important place to overcome and fight it^(24)^. In the institutional dimension, it is capable of producing and/or maintaining the vulnerability of individuals and social groups made vulnerable by racism^(17)^.

School silencing in the face of discrimination is associated with the ideological presence of “Brazilian racism”, that defends equality of the Brazilian mixed-race population, denying differences and racism itself. This way, non-confrontation is due to the phenomenon of naturalization, where suffering is translated as something specific of the child/adolescent, without actually delving into contexts, collectivities, and racial constructions^(25)^.

The field of education is extremely important for the confrontation and fight against racism, attitudes of discrimination, prejudice, and exclusion. It shall be recognized and receive investment from *CAPSij*, as a partner in the construction of transformations in the oppressive reality to which black children and adolescents are subjected, through an education that provides the construction and affirmation of a resistant and positive black identity, through its transforming and liberating character, in search for a solidary and fair society^(23)^.

When a child mentions not accepting their black stereotypical appearance and features (skin color, nose, hair and/or mouth), this reveals the impact of the personal/internalized dimension of racism in the construction of identity, because when they realize that everything that concerns them is undervalued, they experience their body non-acceptance^(17)^. Investigators highlight that the prevalence of common mental disorders in adolescence in a public school was 52.2% and, of these, 78.7% self-declare themselves as black^(26)^. In addition, black children and adolescents are exposed to the risk of self-mutilation and suicidal ideation^(26)^. The Ministry of Health reported that among suicides, the proportion was 55.4% black to 39% white. When adolescents (10–19 years old) and young people (20 to 29 years old) are selected, for every 100 suicides among white young people, there are 145 suicides among blacks^(27)^.

Black children and adolescents hardly find racial representation in the media or activities involving black people, characters and dolls, in places of power and prestige; investing in interventions in the *CAPSij* scenario, on its walls, practices and proposals is of fundamental importance to expand self-esteem, appreciation, and empowerment of the black population who is followed by the service, focusing on the personal and interpersonal dimension of racism^(17)^. The media plays a role in building the identification of the subject. For the black community, negative stereotypes are always reinforced, emphasizing violence and the place of submission in society^(28)^. Through the myth of racial democracy, whiteness takes care of its place of social privilege, builds an unattainable image as perfection, internalizing a model of white and Eurocentric culture^(28)^.

In the construction of identities, reference technicians use objects, toys, events, images and black public figures to develop the work of representativeness in singular and collective accompaniments. The identification of the black child shall take place through ludic activities, through games to promote child development, considering each child as a subject^(29)^. The importance of having professionals in health services who are similar to the children attended is highlighted:

*Recalling the ethical and political commitment existing in the fact of always considering contact with the subject – in this case, children, it is essential to offer adequate conditions for the global development of subjectivity, with ethnicity or its phenotypic representations being very important elements of this work. In this regard, children should be able to experience in their group and institutional experiences the possibility of an adequate and also varied ethnic positioning, with other ethnicities, allowing the reach of the concept and the condition of full humanity* ^(29)^.

The fragility of the reports in the medical records is pointed out as a result. Their role is to allow the continuity of care processes, making racism visible as violence and formulating strategies for combating and coping with it. It is necessary to create time and meaning for this action in the teams’ work process. In general, these weaknesses are institutional:

*With regard to the causes related to the main non-conformities of reports in medical records, we can observe a lack of attention and interest from the worker in recording the care cycle, the work overload, the deficit of human resources. The lack of knowledge of the legality and the lack of qualification were elucidated as important causes of non-conformities for the optimization of records in medical records* ^(6)^.

The qualified, periodic, procedural, and analytical collection of the race/color question will contribute to *CAPSij*’s work so that children, young people, and family members can recognize themselves as black, thus indicating that to be black is to become black^(30)^.

Brazilian-style racism hides its form and acts on naturalization, anchored in the myth of racial democracy, denying the existence of inequalities given miscegenation and supposed equality^(25)^. Thus, actions of inclusion to face the psychosocial and institutional effects of racism within the *CAPSij* are required, such as the action of “*aquilombação*” of the service, decolonizing knowledge and practices. “*Aquilombar”* is defined as:

*rescue of traditional knowledge; the decolonization of therapeutic practices; bringing African and diasporic theories and teachings from Latin America and the Caribbean into the context of psychiatric reform, fighting epistemicide; the resignification and naming of therapeutic practices in a racialized way, understanding that they serve different singularities; bringing health services closer to movements and collectives that aim at racial equity (…)* ^(15)^.

The rescue and appreciation of history, the access to black culture are described by TRs in the mental health service, in their empowerment actions, interventions, and manifestations, in the intersectoral work, in the relationships and integrations with cultural, art, and education facilities. Thus, knowing oneself, culturally belonging to a social group, empowering oneself with his/her history is a way of exercising autonomy^(30)^.

## CONCLUSION

The universalization of care did not overcome the inequities experienced by the black population. There is difficulty in implementing the health policy for the black population at the intersection of mental health, due to the denial of racism and lack of knowledge about how to detect and face it. It is the role of community mental health services, in a network, to create mechanisms to increase the contractual power of people in social spaces, since by not discussing racism, invisibility is created and this barrier affects contractuality.

The limitations of the study are the time for collection, the saturation of interviewed technicians, only one *CAPSij*, which does not demonstrate the reality of the Psychosocial Care Network. It is necessary to study the question of mental health care quality in the race/color segment of children and adolescents with larger samples in the context of *RAPS*. More studies are recommended and they shall qualify practices from the perspective of rights.

The race-color question as a social marker is able to guide the *PTS* of black children/adolescents when understood as a category of analysis in the fight against racism. Inclusion actions were aligned in the approach to discrimination and constraints, regardless of the age of the child/adolescent, such as reporting in the medical records, notifying violence, working on representation in the development of black identity, weaving intersectoral networks.

Finally, the actions of inclusion, when they allow a social place of right, potentiate care and autonomy of children/ adolescents with mental health problems. To democratize the institutions, the participation and accountability of children and adolescents, their families and the community shall be added to the technical objectives of the work at *CAPSij*.

## ASSOCIATE EDITOR

Rosa Maria Godoy Serpa da Fonseca
